# Suspicious conspiracy theories

**DOI:** 10.1007/s11229-022-03602-4

**Published:** 2022-05-31

**Authors:** M R. X. Dentith

**Affiliations:** 1 Center for International Philosophy, Beijing Normal University at Zhuhai, 18 Jinfeng Rd, Zhuhai, 519087 Guangdong China; 2 School of Philosophy, Beijing Normal University, 19 Xinjiekouwai St, Beijing, 100875 China

**Keywords:** Conspiracy, Conspiracy theory, Conspiracy theorist, Epistemology, Suspicions, Social epistemology, Warrant

## Abstract

Conspiracy theories and conspiracy theorists have been accused of a great many sins, but are the conspiracy theories conspiracy theorists believe epistemically problematic? Well, according to some recent work (such as Cassam Quassim, Keith Harris, and M. Guilia Napolitano), yes, they are. Yet a number of other philosophers (myself included) like Brian L. Keeley, Charles Pigden, Kurtis Hagen, Lee Basham, and the like have argued ‘No!’ I will argue that there are features of certain conspiracy theories which license suspicion of such theories. I will also argue that these features only license a limited suspicion of these conspiracy theories, and thus we need to be careful about generalising from such suspicions to a view of the warrant of conspiracy theories more generally. To understand why, we need to get to the bottom of what exactly makes us suspicious of certain conspiracy theories, and how being suspicious of a conspiracy theory does not always tell us anything about how likely the theory in question is to be false.

## Introduction

When it comes to the scholarly discussion on conspiracy theories, philosophers have (with a few exceptions) argued that there is nothing generally or inherently irrational or suspicious about belief in such theories.

Basham (2001); Coady (2003); Hagen (2018); Keeley (2007) and Pigden (1995), and I (2021) have all advanced arguments as to why we should take conspiracy theories seriously. These philosophers understand conspiracy theories as putative explanations which concern the existence of conspiracies: activities undertaken in secret by two or people towards some end. That is, conspiracy theories are theories–in the explanatory sense—about conspiracies.^1^ The aforementioned philosophers have argued that there is nothing inherently irrational about belief in such theories when properly understood. Rather, such theories are as good or bad as the evidence which counts for or against them. As such, they have adopted what has come to be called in *conspiracy theory theory* (the academic study of conspiracy theory) ‘particularism’: the thesis that we should evaluate individual or particular conspiracy theories on their evidential merits (or demerits), rather than make crude generalisations about the class of things labeled as ‘conspiracy theories.’^2^

Some philosophers have demurred from the particularist position. Cassam Quassim, for example, has taken particularists to task for being ‘conspiracy apologists’ (2019). Keith Harris (2018) and M. Guilia Napolitano (2021) refer to conspiracy theories as *epistemically* problematic. Harris argues that, generally, there are epistemic errors heavily implicated in the activity of conspiracy *theorising*, which makes such theorising epistemically suspect (2018). M. Giulia Napolitano has gone on to accuse particularists of conceptually re-engineering the notion of conspiracy theory in a way which is ‘neither warranted nor fruitful (2021, p. 85).’

We might think that these critics of particularism–the generalists (who argue that, generally, we have grounds for something like a *prima facie* suspicion of conspiracy theories)–have a point. Perhaps conspiracy theories are putative explanations about the existence of conspiracies, but according to philosophers like Cassam, Harris and Napolitano, there really is something suspicious about belief in such theories. Indeed, recent examples of conspiracy theories–like the plethora of COVID-19 conspiracy theories and the rhetoric which led to the January 6 Protests/Insurrection at the US Capitol in early 2021–point to a problem with belief in conspiracy theories generally.

Indeed, much of the generalist literature uses examples such as these (along with belief in 9/11 conspiracy theories which suggest the US government was behind the terror attacks (Cassam , 2016); Flat Earth beliefs (Harris , 2018), Moon Landing Hoax hypotheses (Napolitano , 2021), and the like) to motivate the argument that not only are a great many conspiracy theories *epistemically problematic*, but that this justifies a *prima facie* dismissal of such theories. Much of this work points towards prior research in the social sciences–particularly social psychology—for evidence of the worrying or dangerous nature of conspiracy theories.^3^

This has led generalists like Cassam to eschew the assessment of conspiracy theories on the evidence and, instead, be more interested in talking about how conspiracy *theorists* suffer from the epistemic vice of gullibility. Under his account conspiracy theories are part of a set of *bizarre* views (2016).^4^

M. Giulia Napolitano talks about conspiracy theories as being *absurd*, and argues that particularists have:

[H]arshly criticized researchers with different approaches to the topic for their negative attitude towards conspiracy theories and for ‘pathologizing’ belief in such theories, thus creating a hostile intellectual climate where different research projects on conspiracy theories seem to be talking past each other (2021, p. 85).^5^

Instead, she argues that we should accept that:

[T]he public debate about conspiracy theories assumes that conspiracy theories are fictions that undermine the trust required for the spread of knowledge in our societies, and that belief in such theories is inappropriate (2021, p. 82).

Generalists (both within and outside of Philosophy) do not dispute that conspiracies occur; their ire is reserved for these things called ‘conspiracy theories.’ Even though conspiracies occur, they take it that most conspiracy theories can be treated as *prima facie* unwarranted^6^. The label ‘conspiracy theory,’ then, does not merely refer to some putative explanation about a conspiracy but, rather, concerns (to some extent at least) talk about unwarranted or unfounded speculation about the existence of some conspiracy.

Conversely, particularists often use examples of well-attested to conspiracies–like the pre-ordained verdicts of the Moscow Show Trials of the 1930s, the Atomic Energy Commission covering up the deleterious effects of radioactive fallout in the 1950s, the second Gulf of Tonkin Incident in the 1960s, the Iran-Contra scandal of the 1980s, and the Weapons of Mass Destruction rationale for the invasion of Iraq in the 2000s (to cite just a few examples)–to show that merely accepting ‘conspiracy theory’ as a label which refers to unwarranted claims or unfounded speculation about the existence of conspiracies can have unfortunate social consequences (such as leading people to ignore cases of powerful people conspiring simply because putative explanations of these conspiracies have been labeled as ‘conspiracy theories’).^7^

Indeed, as Basham (2011) and I (2016b) have independently argued, the history of actual conspiracies can play a positive role in determining whether a particular conspiracy theory *now* is warranted.

The history of past conspiracies is not, then, a feature we can necessarily use to show that conspiracy theories ought to be treated with suspicion. That being said, particularists do not necessarily disagree that belief in *some* particular conspiracy theories can be *mad, bad and dangerous*. Even those of us who argue that we cannot *on principle* claim conspiracy theories are generally epistemically defective have labelled some conspiracy theories as problematic because they are *mature* (Keeley 1999), *fantastical* (Räikkä and Basham 2018), feature highly *defectible* conspiracies (Pigden 2018), or are just examples of *recurrent* narratives (Dentith 2016a).

That is, there are many conspiracy theories with features we find *suspicious*, and we typically use such features to say that these particular conspiracy theories need not be taken all that seriously. However, as we will see, we cannot use these features to cast aspersions on the entire class of conspiracy theories.

## Mature conspiracy theories

Brian L. Keeley argues that there is a class of conspiracy theory we are justified in being suspicious of: the *mature* unwarranted conspiracy theory (1999).

Keeley is interested in what our attitude should be towards conspiracy theories which persist despite no positive evidence accruing for them over time. According to Keeley, if a conspiracy theory fails to gain warrant *but continues to persist*, then it becomes *mature*. ‘Maturity’ is like a mouldy cheese, or an old egg; it stinks, and that stench is a reason to treat it with some suspicion.

Such conspiracy theories are suspicious because they have failed to gain adequate evidence in favour of them: they have *matured* but failed to become accepted as part of the conventional wisdom. That is, despite (presumably) being actively researched, no positive evidence has accrued in favour of these theories.

Keeley’s chief example of such a mature conspiracy theory was the Oklahoma City Bombing conspiracy theory that implicated the Bureau of Alcohol, Tobacco and Firearms (AKA the BATF) as being involved in the attack on the Alfred P. Murrah Federal Building in 1995. Other examples might include certain 9/11 Inside Job conspiracy theories, which posit that elements within the US establishment orchestrated the September 11th terror attacks, or conspiracy theories about the thesis vaccines are the primary cause of autism. In each of these cases the conspiracy theories have persisted despite no major breakthrough, which in turns gives rise to the suspicion that perhaps there is not much substance to these theories.

## Recurrent conspiracy narratives

Sometimes, as I have argued previously, we are suspicious of particular conspiracy theories because they resemble a theory we already have reason to think of as suspicious. That is, it is an example of a *recurrent* conspiratorial narrative (2016a).’

Take, for example, conspiracy theories that claim mass shooting events are part of a government-led conspiracy to bring in strict gun control/regulation: every time a new mass shooting event occurs someone posits that the event must have been staged, and thus is just another instance of a false flag event.^8^ Or we can point towards the continued prevalence of anti-Semitic conspiracy theories, in which any bad event is blamed upon the Jewish people.

These theories are recurrent, and thus are the kind of thing we typically treat as suspicious because we have not just seen them before, but we have seen past instances be shown up as unwarranted. These conspiracy theories are, effectively, new examples of mature conspiracy theories which have been repackaged or relabelled, and thus–via their resemblance to an existing mature conspiracy theory–we can say such theories are suspicious.

## Fantastical conspiracy theories

Juha Räikkä and Lee Basham claim some conspiracy theories are so *fantastical* that this licenses some suspicion of them.^9^ As they state:

Some conspiracy theories make claims so fantastical that they go beyond what most people can accept as true. For example, the claim that interdimensional lizard people secretly rule the planet is an extraordinary one, and therefore requires extraordinary evidence. While bizarre conspiracy theories like this are not representative of all or most conspiracy theories, they may spoil the whole, thereby driving people to reject, out of hand, more mundane and more evidenced claims of conspiracy (2018, p. 180).

That is, some conspiracy theories are fantastical in the sense they are so counter to our experience of the world that no sensible person would believe. Conspiracy theories, for example, which posit the existence of interdimensional lizard people, or a world which is simply a flat disc floating in space are the kind of thing most people not only think are in no way plausible, but they are so implausible that they are fantastical. By-and-large, most people do not think interdimensional lizards exist, and the curvature (and thus roundness) of the Earth is easily verified by seeing the horizon and moving towards it. As such, if a theory goes well beyond the available evidence it is fantastical, and so we can treat it with suspicion.

## Defectibility

A recurrent theme when it comes to the criticism of conspiracy theories is the supposed problem of keeping conspiracies secret, especially in the face of active investigations.

Charles Pigden has argued that sometimes we have grounds for a suspicion of particular conspiracy theories which feature conspiracies we think should have come to light if they really existed. He deems this the problem of ‘defectibility:’

A conspiracy is defectible if the costs of defection are low and the rewards of defection are high. A theory has defectibility if the conspiracy it postulates is defectible (2018, p. 209).

Take, for example, the Edward Snowden revelations about the NSA surveillance programme: the potential cost of defection–AKA revealing the surveillance of US citizens–was admittedly high (see, for example, the punishment meted out to Chelsea Manning for her revelations of government secrets), but the rewards for defection/whistleblowing turned out to be higher still. Thus, the secret surveillance of US citizens by their own government was revealed.

The problem of defectibility is this: if the benefit to a member of the conspiracy revealing the conspiracy is higher than the cost of defecting from it, then we should expect someone to at least to act as a whistleblower. Of course, not all conspiracies are *highly* defectible, but where a particular conspiracy theory features a defectible conspiracy and yet, over time, no defects from it, then we have grounds for concern.

We can think of defectibility as a companion analysis to that of maturity. In the same way that an unwarranted conspiracy theory will eventually become *mature* if time passes and no positive evidence accrues for it, a conspiracy will come to suffer from the problem of defectibility if the conspiracy in question remains highly defectible and yet no one defects from it. So, if a conspiracy theory features a highly defectible conspiracy (for example, it is said to have been running for a long time yet no one has defected from it), then this is grounds for treating it with suspicion.

## What the suspicious features of particular conspiracy theories entails

As we have seen, particularist philosophers of conspiracy theory theory have–contra the accusations of some generalists—been concerned that there are features which license some suspicion of conspiracy theories. Often we are suspicious because a preliminary analysis causes us to note that the conspiracy theory has some feature we associate with other theories we find suspicious, or said theory has a feature which makes it unlikely the conspiracy theory can be warranted.

However, these preliminary analyses generate what we might term—at best—*weak* suspicions:

*Weak suspicion*: If a claim has one of the aforementioned features, this can generate in epistemic agents a *limited* suspicion which justifies treating the claim as unwarranted.

Let us call this a type I suspicion. Type I suspicions are weak because whilst they license a certain scepticism of a given theory, they do not tell us the theory is false.

For example, Keeley, in his 1999 takes it that the conspiracy theories about the BATF being involved in the Oklahoma City Bombing are *mature*. Given the short time between the event in 1995 and the drafting, writing and publication of his paper in 1999, we might think it presumptuous to consider the BATF theory mature a mere few years after the event. Consider this: many journalists probably thought for years that Bob Woodward and Carl Bernstein’s 1972 story about the Watergate Hotel break-in was just a poorly evidenced conspiracy theory; sometimes it takes a while for the evidence to come out. That being said, many of us probably considered several of the COVID-19 conspiracy theories which emerged in early 2020 mature by the middle of that year.

A theory’s maturity, then, is simply a guide as to how suspicious we should be of it with respect to both its persistence *and how thoroughly it has been investigated.* Indeed, as Keeley noted back in 1999, given that conspiracy theories concern conspiracies, it is possible that this lack of positive evidence might be due to the conspirators successfully keeping evidence of their conspiracies from us.^10^ This is why the maturity of a given theory tells us that we should be suspicious of it *all things considered* but it cannot tell us that the theory is suspicious-qua-false.

We can say something similar about conspiracy theories which feature *seemingly* highly defectible conspiracies which no one has defected from: the defectibility of a conspiracy is dependent on factors both internal *and external* to the conspiracy.

Imagine, for example, a 9/11 plotter who-in return for clemency or a full pardon–reveals to the Barack Obama administration that former President George W. Bush and his administration were responsible for the events of 9/11. Because the benefit of revealing the prior president’s war crimes will likely lead to a given conspirator being treated leniently, we can say the conspiracy theory *looks* highly defectible. But this assumes that with a change of administration the cost/benefit analysis also changes. What, though, if another Republican had won the election? Or what if you think that whilst Obama was ostensibly a different kind of political leader to his predecessor, the actual machinery of government didn’t change at all. After all, Obama ended up being as hawkish (if not more-so) than W. Bush in the final accounting...

The defectibility of a conspiracy can, then, wax and wane. Revealing to a second term Trump administration that you were central to a White House-led campaign to underplay the seriousness of the COVID-19 pandemic over the course of 2020 would likely harm you rather provide you some benefit. Conversely, if you revealed this to an incoming Biden administration in early 2021, then you would likely would have been rewarded.

Not just that, but some conspirators will be aware that their conspiracies might be prone to defection. Some conspirators might be the kind of people to employ murderous enforcers, to ensure that even under a change of circumstances potential defectors might decide to not risk their lives or those of their loved ones.^11^ As such, a conspiracy theory *appearing* to feature a conspiracy which *looks* like it suffers from the problem of defectibility only gives us grounds to be suspicious of it in a weak sense.^12^ Someone needs to analyse the substance of the conspiratorial claim to see whether the conspiracy really does suffer from the problem of defectibility after all.^13^

Or what about those *fantastical* conspiracy theories? Well, what counts as ‘fantastical’ is often context-dependent. David Icke–the proponent of the conspiracy theory concerning those interdimensional lizard people–is well aware that his theory *seems* fantastical. This is why–when he lectures on the topic–he spends several hours getting to the revelation that behind human history it is reptiles! reptiles! reptiles!^14^

So, whilst we might want to say that interdimensional lizard people are fantastical *because they go beyond our understanding of how we think the world should work*, they are still worth investigating (at least by someone) in order to work out whether they really are counter to our *current* understanding of the world. After all wouldn’t it be interesting if it turned out upon investigation that interdimensional lizard people really were in charge of global politics? Indeed, if it turned out there was something to such a claim, we would probably agree with Icke that something ought to be done about it!

Finally, a theory can *resemble* a recurrent narrative we already think is suspicious, but if it relies on new evidence or novel arguments, then it ought to be analysed afresh.

Here is an example to illustrate this concern: there are numerous conspiracy theories about the state of climate science which suggest that climatologists and members of related fields have perpetuated the allegedly fraudulent science of anthropogenic climate change. These theories are both mature and suffer from a problem of defectibility as, after countless investigations, they have been shown to be wanting.

Yet we can also imagine that tomorrow climatologists–who have been warning us of the danger of anthropogenic climate change for nearly half a century now–might decide to do something drastic. They believe that no government or corporation is doing enough to mitigate the worst of the coming climate crisis, so they decide to do the thing they have been blamed for doing all along: they come together in secret to start exaggerating the evidence for an impending climate collapse, all in the hope that this will cause the public to demand immediate action from their governments!

There is nothing stopping climatologists from doing this, which means it is not impossible that this new theory–which *resembles* a similar but unwarranted conspiracy theory–is warranted. That is, resemblance to a theory we already think of as unwarranted allows us to treat such a theory with suspicion, but not such that we should dismiss it without someone looking into it.

What is the moral here? Well, the fact we find some conspiracy theories *mature*, *fantastical*, an example of a *recurrent narrative*, or that they feature highly *defectible* conspiracies does not tell us that said theories are false. Rather, these features simply tell us that, all things considered, the theory looks suspicious.

Suspicions can, of course, be useful: in a situation where we have little time but a lot of demands on it, we are better off if we can spend our time looking into, say, the more plausible claims about the existence of conspiracies.

We might consider this to be an *economic problem.* Most of us do not have the time or ability to investigate the many conspiracy theories we encounter on a week-by-week–or, it seems increasingly, day-to-day–basis. We need, then, to pick-and-choose our battles and prioritise which theories we ought to focus on now, and which we might investigate *if we have the time or energy later*. So, because we often need to prioritise our precious time, we often have to make do with weak/type I suspicions. However, this should always be on the proviso that when we have the time and energy, we should check to see if our suspicions are vindicated. Or, at least, we should check to see if someone else has done this. After all, only a few people will want to spend the time and effort investigating fantastical theories or recurrent narratives...The prioritisation of our resources is, however, a pragmatic concern, rather than a strictly epistemic issue.

Such investigations will allow us to see if our weak suspicions can, on investigation, be strengthened. As such, we can contrast *weak* suspicions with their *strong* counterparts (type II suspicions):

*Strong/Type II suspicions:* Upon investigation a claim with one of the aforementioned features generates in epistemic agents a strong suspicion that the claim is unwarranted or even false.

Obviously type II suspicions are better than type I suspicions. But generating type II suspicions is often hard because it takes time, effort, and expertise. Type I (weak) suspicions, then, are useful for prioritising our enquiries or investigations. Such suspicions don’t necessarily tell us the view or theory in question is unwarranted in the sense it is false, but they might tell us that a theory without such features ought to be taken more seriously.

### Warrant

Both weak/type I and strong/type II suspicions generate the suspicion that a conspiracy theory is ‘unwarranted.’ One feature of the discussion of how we talk about conspiracy theories in the philosophical literature is the use of the term ‘warrant,’ which we owe to Keeley’s 1999 paper. There he was interested how mature conspiracy theories fail to become warranted over time despite persisting in public discourse. That is, such theories are considered *unwarranted* due to never gaining warrant.

However, a lack of warrant—being *unwarranted*—turns out to be ambiguous. We might mean that:

1. The theory *does not yet have warrant*: we consider it unwarranted because there isn’t enough available evidence to justify belief in the theory, or
2. The theory is clearly false according to the available evidence: we should not believe it, and so it is unwarranted.

Weak or type I suspicions tell us a theory is unwarranted in first sense: the theory has yet to gain warrant. This is why we need to check to see if they resolve into stronger suspicions which tell us the theory is unwarranted in the second, stronger sense. That is, we cannot say that conspiracy theories which are–in the words of Cassam–*bizarre* or–in the words of Napolitano–*absurd*–turn out to be epistemically suspicious unless we commit to a central tenet of particularism: actually checking to see if the bizarre or absurd nature of the theory upon investigation justifies thinking our suspicion of it is a strong or type II one.

This is why we can’t use the features of suspicious *seeming* conspiracy theories to generalise about the suspiciousness of the class of conspiracy theories as a whole. After all–as we have seen–those weak suspicions come with caveats. It is one thing to say a conspiracy theory *with certain features* is suspicious, and thus need not be investigated right now. It is another thing, however, to then generalise from such suspicions to a *prima facie* suspicion of *all* conspiracy theories.

## What to do when people disagree about the features?

One objection to the argument as presented is that people might disagree whether the features in question–whether it be, say, maturity or defectibility–are a problem for a given theory or conspiracy.

For example, whilst some particularists might take it that most ‘Inside Job’ conspiracy theories about 9/11 (i.e. theories that say the US government orchestrated the attacks) suffer from the problem of defectibility, others might not. Whilst not endorsing such a theory, Basham argues that by a back-of-the-envelope calculation, the number of people involved in setting up a controlled demolition would not be anywhere as large as the thousands some sceptics of the controlled demolition theory argue (2016, fn11). That is, you might think that an inside job account of 9/11 would require a lot of people, and thus you might think this makes it all the more likely someone would speak out about it, but someone else might argue it could have been undertaken by a small and committed group of conspirators who have managed to keep quiet about their involvement.

So, what should we do in situations where people disagree on such features? Well, such disagreements might focus:

1. on a particular piece of evidence
2. on the weight of the total evidence, or
3. be the result of people being bad judges of certain kinds of evidence.

Options 1 and 2 speak to the difficulty of assessing a claim of conspiracy. As particularists have argued, part of the problem with assessing any claim of conspiracy in a conspiracy theory is the question of whether there is dis- or misinformation being put out by the conspirators. Both Basham (2018b) and I (2016b) discuss this with respect to just how we might establish the prior probability of conspiracies generally in the political climate the conspiracy theory in question emerges from. Keeley discusses this with respect to why the seemingly unfalsifiable nature of *some* conspiracy theories is a feature, not necessarily a bug, of some of those theories (1999).^15^

Whilst we might like to think we can easily assess evidence in these cases, many of the conspiracy theories we find interesting—Inside Job hypotheses; QAnon accounts; and the like—are often complex theories relying on masses of evidence, and so some disagreement on the weight of some particular piece of evidence, or the weight of certain parts of the available evidence is probably to be expected.^16^

It is also useful to note that type I or weak suspicions–at least in the language used here–are features that *might* generate such suspicions. Not everyone is going to be attentive to these features, or aware of their evidential weight with respect to some theory. That is, sometimes people will disagree simply because they do not notice that the theory in question has a particular kind of feature.

Options 1 and 2 also show why we have to be cautious when making the move from claiming that a weak or type I suspicion will likely resolve into a type II or strong suspicion: diagnosing a conspiracy theory as having one of these features is no guarantee that the theory will turn out to be suspicious in the strong sense. This is why we are still obliged to do the work–when we can–to check to see if they might resolve into strong suspicions.

Now, a theory having multiple features which generate weak suspicions might well be another story: a *mature* theory which features a *highly defectible yet undefected* conspiracy (which is also an example of a recurrent conspiracy narrative) should not be the kind of theory particularists will disagree on...*unless one of them is simply a bad judge of evidence*. We could say that if a conspiracy theory has two or more of the aforementioned features, this will generate in epistemic agents a strong/type II suspicion, which justifies treating the claim as unwarranted.

Such theories will be considered suspicious in a strong sense, since the likelihood a theory would appear to have all those features yet still turn out to be warranted will be incredibly unlikely. That being said, it might still only be unwarranted in the sense there isn’t enough evidence to believe it *now.* Investigation into the claim might still be necessary by someone...

This distinction between type I/weak suspicions and type II/strong suspicions, and what they suggest about the warrant of conspiracy theories, should be applicable to theories more generally. Conspiracy theories turn out to be illustrative of the limit of using suspicious features to judge the warrant of theories generally because of the commonplace dismissal of conspiracy theories (especially given that particularists argue, qua-Pigden, that this dismissive attitude is a modern superstition (2006)). The lessons learnt here, however, should be applicable to theories elsewhere.

## Conclusion

Particularists have, largely, focused on the epistemic question of when belief in a particular conspiracy theory is warranted or unwarranted. Still, as we have seen, much thought has been given to the question of whether some conspiracy theories have features which do, in fact, license a *limited* degree of scepticism. Particularists have not, then, ignored the *potential* social consequences or costs of belief in some conspiracy theories. Rather, the main thrust of the philosophical work in this area has been to take seriously the threat of undetected conspiracies in our polities, since these have known and proven negative social consequences.

Thus, contra Napolitano, particularists have not been talking past other scholars who study conspiracy theory. Rather, they have simply been reminding them that as conspiracies occur, we must remain vigilant, even if there are certain conspiracy theories which turn out to be suspicious.

The preceding analysis also acts as a reply to Cassam: particularists are not engaging in conspiracy apologetics. They have–since the Nineties–been attentive to the problems associated with belief in some conspiracy theories. Rather than apologetics, particularists have been interested in showing why the kind of scepticism we show towards *some* particular conspiracy theories cannot and should not be generalised to *all* conspiracy theories.

What this analysis also shows is that if generalist critiques of belief in conspiracy theories rest upon claims that x, y, or z conspiracy theory is suspicious; therefore conspiracy theories as a class are suspicious, then this likely mistakes type I/weak suspicions for type II/strong ones. That is, such a generalism will often make the mistake of overgeneralising from particular cases to conspiracy theories more generally. Thus, contra Harris, we cannot move from particular cases of suspect conspiracy theories to a judgement on conspiracy theorising generally.^17^ Rather, these features allow us *at best* to prioritise the investigation of some conspiracy theories at the expense of others. Diagnosing theories as having these features provides some solution to the economic or pragmatic problem of most of us not having enough time, energy, or the right expertise to investigate them. When it comes to appraising the wide variety of conspiracy theories we *seem* to encounter on an increasingly frequent basis these days, these features can be of benefit...as long as we do not overstate our confidence in them.

## Footnotes

1 For further discussion how our choice of which definition to use affects the kind of analyses we perform on these things called ‘conspiracy theories’ see my 2018a

2 There is some debate in this literature about how we should further refine the definition of what counts as a ‘conspiracy theory.’ Some particularists argue such theories are necessarily sinister in nature (thus, no ‘conspiracies of goodness’), are only viable if the number of participant conspirators is relatively small (no all-embracing conspiracies), or that they must be in conflict with some official theory. However, particularists by-and-large do not think that these other (debatable) features single out conspiracy theories as necessarily unwarranted.

3 For some recent examples of such studies, see Karen Douglas’ 2021, Stephan Lewandowsky’s 2021, and Ashraf Sadat Ahadzadeh, Fon Sim Ong, and Shin Ling Wu’s 2021.

4 In subsequent work Cassam has moved away from a vice epistemology-based assessment of what is wrong with conspiracy theorists to a political argument where he defines a special category of conspiracy theories, the ‘Conspiracy Theory.’ These capitalised examples, according to Cassam, are contrary in nature, put forward by amateurs, and embody a pre-modern view of the world, which Cassam argues makes them a form of right-wing propaganda 2019. For a critique of Cassam’s more recent work on the topic, see Hagen’s (in press).

5 Napolitano cites a debate between social scientists and philosophers, but fails to note not only the harsh criticisms made by the social scientists (Dieguez and Bronner , 2016; Wagner-Egger et al. 2019), but that they also falsely attributed positions to their critics; see, for example, my and Martin Orr’s 2017 and Hagen’s 2017 for details.

6 See, for example, Karl Popper 1969, van Prooijen et al. (2018), and Lewandowsky et al. (2018). Indeed, some generalists (like Swami et al. (2014) and Daniel Pipes, 1997) argue that conspiracy theories are just false by definition.

7 For more discussion about the perils of the label ‘conspiracy theory’ see Husting and Orr (2007), Pelkmans and Machold (2011), Bjerg and Presskorn-Thygesen (2016), and McKenzie-McHarg and Fredheim (2017).

8 The term ‘false flag’ comes from wartime efforts to make it look as if an event or attack was committed by another party by using their flag rather than the attackers’ own.

9 The context of Räikkä and Basham’s argument is with respect to why some people are not particularists. In a footnote they state: Our working assumption here is that people do not think that *most* conspiracy theories are insane merely because *some* conspiracy theories–for instance theories that concern the alleged actions of the Antichrist–are insane (2018, fn10).

10 This kind of claim will turn out to be an auxiliary hypothesis, and so can appraised as warranted or unwarranted. If, for example, disinformation is being produced by the conspirators, then–as I have argued (2019)–we can appraise the warrant of that auxiliary hypotheses when it comes to assessing the conspiracy theory as a whole.

11 Like the aforementioned discussion of disinformation, such auxiliary hypotheses can be assessed as part of the general assessment of the particular conspiracy theory.

12 As I argued with Orr, a given conspiracy theory can appear to have a large number of conspirators, and thus suffer from a particular problem of defectibility (the ‘Surely someone in the conspiracy will trip up and become an inadvertent whistleblower’ kind). However, as we argue, if the conspiracy is organised in the right way, only a few conspirators might know the full extent of what is going on, and so the conspiracy will not be *as* defectible as it might appear on first glance 2018.

13 One salient difference between maturity and defectibility is a defectible conspiracy might become utterly undefected, whilst it may still remain possible that evidence for a mature conspiracy theory could come to light. After all, eventually all the people involved in a given conspiracy will die, which means that even if it were highly defectible, no one will be able (at that point) defect from it. However, for some mature conspiracy theories it will always remain possible that new evidence could come to light which would show it is warranted after all.

14 I have attended at least two of these talks by Icke by time of publication, and they both were over eight hours long. Icke at least attempts to try and make his conclusions seem not that extraordinary in the grand scheme of things.

15 As Keeley argues, a salient difference between a claim of conspiracy and, say, a claim in the sciences is that electrons do not lie about their superposition (1999). It is not irrational to posit that conspirators will at least try to cover up elements of either their existence or their machinations.

16 This is a topic I have discussed at length elsewhere (2019).

17 It might be possible to construct a generalist critique of conspiracy theory which avoids such a move, but it would be akin to the kind of ‘defeasible generalism’ explored by Patrick Stokes (2018) rather than, say, that of Cassam. But we should note that Stokes’ motivation for such a middle ground between generalism and particularism seems overstated: particularists—as shown–have paid attention to the *ethics of belief* when dealing with the potential social consequences of belief in conspiracy theories (suspicious or otherwise). See, for further examples, the responses to Stokes penned by Basham (2018a), Pigden (2018), and myself (2018b) on why particularists need not extol all conspiracy theories in the process of treating them seriously.
